# Concurrent use of alcohol and cocaine: which is the best drug choice?

**DOI:** 10.1192/j.eurpsy.2022.353

**Published:** 2022-09-01

**Authors:** H. Becerra Darriba

**Affiliations:** Osasunbidea - Servicio Navarro de Salud, Psychiatry - Centro De Salud Mental De Tudela, Tudela, Spain

**Keywords:** nalmefene, alcohol, cocaine, disulfiram

## Abstract

**Introduction:**

Patients with comorbid cocaine and alcohol dependence have a worse prognosis with lack of adherence to follow-up and treatment, frequent psychosocial problems, and higher rates of relapse [1]. Concurrent use of both substances produces cocaethylene, which is associated with more toxicity than cocaine alone [2].

**Objectives:**

To determine the efficacy of disulfiram compared to nalmefene in the treatment of comorbid cocaine and alcohol use.

**Methods:**

A quasi-experimental open study was designed on 41 outpatients, with a follow-up of at least 1 year at the Mental Health Unit, aged between 18 and 65 years, diagnosed with cocaine and alcohol dependence (ICD-10). A minimum simultaneous weekly consumption of 2 grams of cocaine and 12 SD (Standard Drink) of alcohol during the month before, described by self-records was established. Treatment with oral disulfiram 250mg/day was assigned to 21 patients, and with oral nalmefene 18mg/day to 20 individuals. Observation period was for 6 months. Urinalysis and alcohol breath test were carried out twice a week. Abstinence was defined by obtaining negative results for at least 4 consecutive weeks. Statistical analysis were performed using SPSS v21.0 (significance p<0.05).

**Results:**

61.9% of patients treated with disulfiram achieved a minimum of 4 consecutive weeks of abstinence from cocaine and alcohol, compared to 40% in the nalmefene group (χ²=1.188; gl=1; p=0.276). There were no significant differences.

**Conclusions:**

Disulfiram or nalmefene monotherapy seems clinically ineffective or insufficient in reducing the combined use of cocaine and alcohol. Further research is needed to assess the effect of both drugs simultaneously.

**Disclosure:**

No significant relationships.

